# Exploring the Knowledge and Preventive Practices on Isolation Precaution and Quarantine Among Health Care Workers in Ondo State, Nigeria

**DOI:** 10.5334/aogh.2454

**Published:** 2019-05-22

**Authors:** Wasiu Olalekan Adebimpe, Demilade Olusola Ibirongbe

**Affiliations:** 1 Department of Community Medicine, University of Medical Sciences, Ondo, NG

## Abstract

**Background:**

Since the outbreak of Ebola and Lassa Fever in many West African countries, infection prevention has become big business in hospitals and health care settings in affected countries. Emphasis has shifted from the routine prevention of cases to rapid identification of infection symptoms and timely initiation of transmission-based precautions in order to eliminate unnecessary exposure for health care staff, hospital visitors, and other patients.

**Objectives:**

The objective of this study was to explore the knowledge and preventive practices on isolation precaution (IP) and quarantine among health care workers in selected health facilities in Ondo State in southwestern Nigeria.

**Methods:**

Explorative cross sectional study among 800 health care workers in health facilities in Ondo State Nigeria, selected using multistage sampling method. Research instruments used were semi structured self administered pretested questionnaires. Data was analyzed using the SPSS software version 23.0.

**Findings:**

Mean age of respondents was 34.5 ± 8.2 years, 144(18.0%) and 150(18.8%) had heard about isolation precaution and quarantine respectively. Only 82(10.3%) and 118(14.8%) had taken part or witnessed isolation or quarantine procedure respectively. While about 64.6% had good mean knowledge scores of universal precaution, only 7.6% and 10.8% had good knowledge score of isolation precaution and quarantine respectively. Gender, occupation and number of years in practice were statistically significant as associated with knowledge scores of IP and quarantine (p < 0.05). Predictors of good knowledge of IP and quarantine were being female and having spent more than 3 years in service as a health care worker.

**Conclusion:**

Poor knowledge of isolation precaution and quarantine was reported among studied respondents. Building the capacity of all health workers on this subject matter would foster this culture of reduction of transmission of infectious disease within and across our hospital settings.

## Introduction

At a time when the West and Central African regions are still battling some diseases, such as the outbreak of mostly Ebola Virus Disease and Lassa Fever, health systems are saddled with the responsibility of doing all it can to contain the epidemic and prevent future occurrences. Beyond the usual concept of universal precaution, further precautionary measures are essential if health care workers are to win the fight against infective microorganisms [1]. Such procedures should be capable of preventing healthcare associated infections, and improve clinical outcomes, reduce costs associated with medical care and ensure an effective use of available infection prevention and control resources.

Though predictors of compliance with universal safety precautions and occupational accidents among health workers are often unreported, the knowledge, attitude and practices of universal precautions among health care workers have been largely studied. Since prevention of healthcare-associated infections may not be 100% possible even in developed countries, it is important to beam our searchlight on those cases of patients who are suspected to be or are already infected towards preventing them from infecting others (source isolation), and/or preventing susceptible patients from being infected (protective isolation), through barrier nursing, segregation mechanisms and mechanical ventilation.

Studies have reported insufficient knowledge of isolation precautions among HCWs [2,3,4]. Among health care workers, physicians have been reported to have a tremendously low level of attitude [2]. In some situations, making a choice of disinfection may be difficult even after consideration of the categories of risk to patients. More worrisome is the culture of quarantine and isolation of the sick, which is alien to Nigerians, and which may require some legal framework and backing for health care workers to implement. Despite the popularity, wide availability and accessibility to infectious disease hospital and tuberculosis (TB) clinics, which are a high risk area due to the nature of the clients being managed and the infectivity of the implicated microorganism, data on isolation precautions and public health measures such as disinfection and quarantine are scarce. In addition, TB and related infection control is an essential, but often overlooked, component of a comprehensive infection control program in developing countries like Nigeria.

Towards uncovering the methodologies to adopt in preventing possible health care associated infections, it is important to determine what knowledge the health care workers have and their practice of isolation precautionary measures, disinfection and quarantine. The objective of this study was to explore the knowledge and preventive practices on isolation precaution and quarantine among health care workers in selected health facilities in Ondo State in southwestern Nigeria.

## Methodology

**Study Area:** The study was carried out in Ondo State with a population of about 484,798 people according to a recent projection of the 2006 national census [5]. The prevalence of TB in the State was not formally documented, while most TB control programmes are under the National TB Control programme. For ease of accessibility, a significant number of activities in TB and TB/HIV care has been decentralized to the Primary Health Care level in addition to those being managed at higher health care levels. Wards in post primary health care settings are avenues to contact patients suspected, or confirmed, of having communicable diseases necessitating isolation precautions and quarantine procedures.

**Study Design:** Health facility based exploratory descriptive survey.

**Study Population:** Health care workers in selected TB care health facilities, infectious disease clinics and selected hospital ward in Ondo State, Nigeria. Eligible health care workers should have been working in his or her unit or health facility for at least six months.

**Sample size:** Using the Leslie Fishers formula for the calculation of sample size for single proportion among a population less than 10,000, and a 14% prevalence rate from a previous study on TB preventive practices among health care workers [6], a sample size of 754 was calculated. This was rounded up to 800 to account for attrition and non-response.

**Sampling technique:** A multistage sampling technique was employed in sample selection. In Stage 1, two out of three senatorial districts in the state were selected through simple random sampling, employing simple balloting. In Stage 2, five local government areas (LGA) were selected per senatorial district through simple random sampling, employing simple balloting. In Stage 3 at clinic level, a list of TB (and TB/HIV clinics) from all eligible health care facilities per LGA was made, six were selected from the list through simple random sampling, employing simple balloting. Also in Stage 3 at ward level, the two infectious disease wards in the state, one TB and one infectious disease ward in each of the two general hospital and only teaching hospital were also selected through simple random sampling, employing simple balloting. Questionnaires were equally allocated per senatorial district and LGA and proportionately allocated per clinics and health facility wards based on the number of eligible respondents per results of these sampling techniques. In Stage 4, all eligible health care workers that met the inclusion criteria and who gave consent to participating in the study were recruited into the study.

**Study instrument:** A semi structured self administered questionnaire with inputs from the WHO guidelines on IPC was used to collect data. The instrument was pretested among 20 TB clinic and ward care workers in a conveniently selected TB health facility in Oyo State. The instrument was also validated by the State epidemiologist and a nationally certified TB control medical officer based in Ondo state. Data collection was carried out with the assistance of trained medical and nursing students. Selected health care workers who were on leave or off duty were appropriately reached through a destination follow up. Data collection took a period of about one month to complete.

**Ethical approval:** Ethical approval to conduct the study was obtained from the ethics review committee of the Ondo State Ministry of Health. Permission was obtained from the heads of selected health facilities while individual respondents gave a written informed consent towards participating in the study.

**Data analysis:** Data analysis was carried out using the SPSS software version 17.0 after data cleaning, double entry and checking for outlier values. Data were presented as charts and tables. Questions related to knowledge was scored accordingly with a score of 1 given to right knowledge for those with “Yes” or correct response; and a score of 0 given to wrong knowledge for those with “No” response. The total score of knowledge was computed and the mean score was determined. Respondents with scores equal to and above the mean were classified as having adequate knowledge while those below the mean score were classified as having inadequate knowledge. The responses from attitude and practices were scored in a similar way. Bi-variate analysis was done using the Chi-squared test, while binary logistic regression showcased association between the major outcome variables and some selected variables, most especially socio-demographic. Level of significance was considered at ρ ≤ 0.05.

## Results

Mean age of respondents was 34.5 ± 8.2 years with age group 21 to 30 years having the highest number of respondents 284(35.5%), 518(64.7%) were female, 306(38.3%) have one to three years of practice, 178(22.3%) were nurses, 498(62.2%) work in HIV/TB clinics (Table 1).

**Table 1:** Socio-Demographic Characteristics of Respondents (n = 800).

Variable	Frequency	Percentage

**Age in years (Mean age 34.5 ± 8.2)**		
1–20	24	3.0
21–30	284	35.5
31–40	324	40.5
41–50	168	21.0
**Gender**		
Male	282	35.3
Female	518	64.7
**Marital Status**		
Married	508	63.5
Single	252	31.5
Divorced	24	3.0
Widowed	12	1.5
Others	4	0.5
**Years of practice**		
<1 years	120	15.0
1–3 years	306	38.3
4–10 years	242	30.3
>10 years	132	16.5
**Occupation**		
Doctor	36	4.5
Nurse	178	22.3
Scientist	200	25.0
Lab. Scientist	234	29.3
Health attendant	152	19.0
**Type of facility**		
TB only	302	37.8
TB/HIV/IDH clinics	256	31.9
Wards	242	30.3

**Figure 1:**
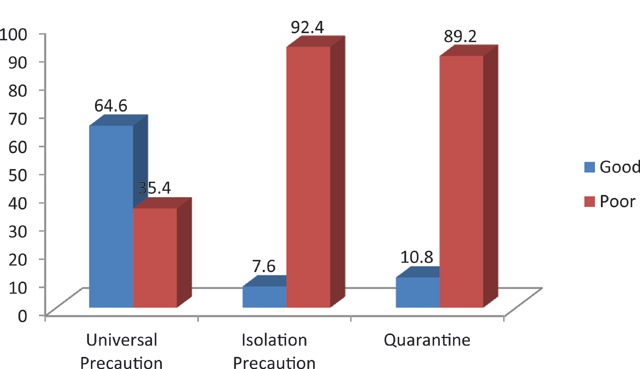
Knowledge scores of UP, isolation precaution and quarantine.

According to Table 2, 726(90.8%) said they were familiar with hospital acquired infection guidelines, 440(55.0%) said they have been trained on IPC, 629(78.6%) said they have read the IPC guidelines, though only 400(50.0%) claim to have a copy of the guidelines. About 64.6% had good mean knowledge scores of universal precaution regarding IPC according to Figure 1.

**Table 2:** Knowledge of Universal Precaution.

Variable	Frequency (n = 800)	Percentage (%)

Infection can be transmitted by medical equipment such as syringes, needles, catheters, stethoscope, thermometers etc.		
Yes	776	97.0
No	24	3.0
Standard precaution includes soaking instruments in an antiseptic solution for up to 36 hours		
Yes	578	72.3
No	222	27.8
Standard precautions apply to all patients regardless of their diagnosis		
Yes	732	91.5
No	68	8.5
I am familiar with hospital acquired infection guidelines		
Yes	726	90.8
No	74	9.2
Policies and procedures for infection control should be adhered to at all times		
Yes	756	94.5
No	44	5.5
Aware that facility (or SMoH) has guidelines on IPC		
Yes	525	65.6
No	275	34.4
Have read the guideline on IPC		
Yes	629	78.6
No	171	21.4
Have a copy of the IPCF guideline		
Yes	400	50.0
No	400	50.0
I have been trained on IPC		
Yes	440	55.0
No	360	45.0
I have up to date knowledge on IPC		
Yes	412	51.5
No	388	48.5

Table 3 shows that 144(18.0%) have heard about isolation precaution, 54(6.7%) said all organisms are isolated in the same way, only 64(8.0%) could list the types of isolation, 142(17.8%) said personal protective equipment (PPE) are indicated during isolation, only 82(10.3%) have taken part in isolation before, 116(14.5%) could list three diseases where isolation is indicated. Eventually only 7.6% had good knowledge score of isolation precaution (Figure 1).

**Table 3:** Knowledge of isolation precaution and quarantine (Yes option only).

Variable	Frequency	Percentage

Have heard about isolation precaution	144	18.0
Isolation means segregation in a single room	136	17.0
Isolation means confining patients in a single room	130	16.3
Could list the types of isolation	64	8.0
Quarantine and isolation are the same	104	13.0
Quarantine is a precautionary measures	115	14.4
Physical protection include barrier nursing, mechanical, ventilation, segregation	110	13.8
All organisms are isolated in same way	54	6.8
Source isolation is to prevent infected patients from infecting others	150	18.8
Protective isolation: means preventing susceptible patients from being infected	142	17.8
PPEs are indicated during isolation	142	17.8
Have taken part in isolation before	82	10.3
Could list 3 diseases that isolation is indicated	116	14.5
Have heard about quarantine	150	18.8
Quarantine apply to sick person	138	17.3
Only suspected cases should be quarantined	138	17.3
Quarantined is done until incubation period is over	142	17.8
Have witnessed quarantine before	118	14.8
Could list 3 quarantinable diseases	138	17.3

Table 3 also shows that 150(18.8%) have heard about quarantine, 118(14.8%) have witnessed quarantine before while 138(17.3%) could list three quarantinable diseases. Eventually 10.8% had good mean knowledge score of quarantine (Figure 1). Table 4 shows that there was a statistically significant association between knowledge score of isolation precaution and gender (p 0.001) and occupation (p 0.001), while similar association exists between mean knowledge score of quarantine and occupation (p 0.001) and number of years in practice (p 0.003).

**Table 4:** Association between isolation precaution, quarantine and some selected variables.

Variable	Bivariate analysis				Binary logistic regression			

	Knowledge score of isolation precaution		X^2^ value	P value	Odds Ratio	95% CI		P value

	Poor n(%)	Good n(%)				Lower	Upper	

Sex			14.176	0.001	3.9038	1.8288	8.3328	0.0000
Male*	274(97.2)	8(2.8)						
Female	465(89.8)	53(10.2)						
Number of years in practice			1.110	0.292	1.4111	0.7415	2.6853	0.1500
<3 years*	620(92.8)	48(7.2)						
4 and above	119(90.2)	13(9.8)						
Occupation			1.904	0.001	0.0177	0.0069	0.0450	0.0000
Nurses*	122(68.5)	56(31.5)						
Others	617(99.2)	5(0.8)						
**Variables**	**Knowledge of quarantine**		**X^2^ value**	**P value**	**Odds Ratio**	**95% CI**		**P value**

	**Poor n(%)**	**Good n(%)**				**Lower**	**Upper**	

Sex			1.063	0.303	1.2899	0.7942	2.0949	0.1529
Male*	256(90.8)	26(9.2)						
Female	458(88.4)	60(11.6)						
Number of years in practice			9.100	0.003	2.1720	1.2994	3.6307	0.0024
<3 years*	606(90.7)	62(9.3)						
4 and above	108(81.8)	24(18.2)						
Occupation			1.996	0.001	0.0407	0.0228	0.0728	0.0000
Nurses*	108(60.7)	70(39.3)						
Others	606(97.4)	16(2.6)						

Female respondents were about four times more likely to have a good mean knowledge of isolation precaution compared to male respondents and this observation was found to be statistically significant (OR = 3.9, 95% CI = 1.8288 – 8.3328, p = 0.001). Respondents who have spent more than three years in practice are 1.4 times more likely to have a good knowledge of isolation precaution compared to those who have spent less than three years and this observation was found not to be statistically significant (OR = 1.4, 95% CI = 10.7415 – 2.6853, p = 0.1500).

Female respondents were about 1.3 times more likely to have a good mean knowledge of isolation precaution compared to males, and this observation was found not to be statistically significant (OR = 1.28, 95% CI = 10.7942 – 2.0949, p = 0.1529). Respondents who have spent more than three years in practice were twice as likely to have a good knowledge of isolation precaution compared to those who have spent less than three years, and this observation was found to be statistically significant (OR = 2.1, 95% CI = 1.2994 – 3.6307, p = 0.002). Thus determinants of good knowledge of isolation precaution and quarantine were being a female health care worker and spending more than three years in practice.

## Discussions

It was largely reported that the practice of infection prevention programs in our health facilities is inadequate in terms of knowledge [7] and compliance with universal safety precautions [8], and this would only contribute to the burden of healthcare-acquired infections [8].

In this study, majority of respondents are familiar with IPC guidelines though about half claimed to have seen a copy. This disagreed with a local study in which majority are aware of universal safety precautions [7]. When health care workers have a copy of IPC guidelines, it is likely that they have read it which in turn may translate to good awareness and knowledge about universal safety precautions. IPC guidelines stipulate the standard operating procedure to follow in order to practice the procedures of isolation and quarantine within health facilities and communities.

In our study, less than one fifth of our respondents were aware of SIP. This is very low when compared to another study in which about half were aware of SIP [9]. Less than one tenth of our respondents had good knowledge of SIP, but about half had good knowledge in a comparative study [9]. In yet another study, overall median knowledge and attitude scores toward standard precautions were above 90%, but median practice score was 50.8% [10].

Poor awareness and poor knowledge of SIP and quarantine reported in this study could be a result of a lack of availability for regular training of health care workers on SIP, the fact that many health facilities do not have IPC guidelines and because PPEs may not be available in a significant number of health facilities. This would, however, lead to increase in the prevalence of health care associated infections and quarantinable diseases.

In this study, female respondents were about four times more likely to have a good knowledge of isolation precaution compared to males. This supports a finding from another study in which females were found to have higher mean scores in knowledge and compliance, with statistically significant differences (*P* < 0.05) [11]. This observation may be connected with the fact that nurses (who are mostly females) work more on the wards and are more likely to come in contact with patients on the ward and so witness procedures that would require isolation and quarantine procedures compared to other cadres of health care workers.

In this study, there was a statistically significant association between knowledge score of isolation precaution and occupation (p 0.001), while similar association exists between knowledge score of quarantine and occupation (p 0.001) as well as number of years in practice (0.003). Though professional cadre was statistically significant associated with knowledge of SIP in our study (with p < 0.05), professional cadre was not a predictor on multivariate analysis. This disagrees with a study in which most nurses (90%) were found to have good knowledge of isolation precautions, though only 65% of nurses reported good compliance with isolation precautions [12]. It also disagreed with another study [11] in which there were statistically significant differences in mean scores of knowledge and compliance between different specialties and academic levels. The implication of our finding is that rather than concentrating solely on nurses, who are health care workers that are more likely to be in more contact with patients on the wards and are supposed to have better knowledge of SIP and quarantine, efforts should be geared towards training all health care workers on IPC irrespective of their cadre. This could be formal training or on the job training in order to significantly step down the menace of healthcare associated infections (HAIs) in studied health facilities.

In conclusion, good knowledge and strict compliance with SIPs was no doubt the best bet for saving patients and health workers from acquiring and spreading HAIs in healthcare settings [13,14]. However in our study, awareness and knowledge of both SIP and quarantine was generally low. This calls for capacity building in form of formal training and on the job training for health care workers. A limitation of this study was the dearth of local literatures on quarantine and SIP, which make comparison of results with other studies a bit difficult. Authors have tried to use available citations within their reach.

## References

[1] Thu TA, Anh NQ, Chau NQ and Hung NV. Knowledge, attitude and practices regarding standard and isolation precautions among vietnamese health care workers: A multicenter cross-sectional survey. Intern Med. 2012; 2: 115.

[2] Kuzu N, Ozer F, Aydemir S, Yalcin AN and Zencir M. Compliance with hand hygiene and glove use in a university-affiliated hospital. Infect Control Hosp Epidemiol. 2005; 26: 312–315. DOI: 10.1086/502545 15796286

[3] Apisarnthanarak A, Babcock HM and Fraser VJ. Compliance with universal precautions among medical students in a tertiary care centre in Thailand. Infect Control Hosp Epidemiol. 2006; 27: 1409–1410. DOI: 10.1086/509857 17152044

[4] Sofola OO and Savage KO. Assessment of the compliance of Nigerian dentists with infection control: a preliminary study. Infect Control Hosp Epidemiol. 2003; 24: 737–740. DOI: 10.1086/502122 14587933

[5] National Population Commission [Nigeria]. Nigeria Demographic and Health Survey Calverton, MD: National Population Commission and ORC Macro; 2016.

[6] Araoye MO. Research methodology with medical statistics for health and social sciences Ilorin, Nigeria: Nathadex Publishers, 3 2003, 2004; 121–122.

[7] Adebimpe WO. Knowledge, attitude, and practice of use of safety precautions among health care workers in a nigerian tertiary hospital, 1 year after the ebola virus disease epidemic. Ann Glob Health. 2016; 82(5): 897–902. DOI: 10.1016/j.aogh.2016.07.004 28283144

[8] Askarian M, Aramesh K and Palenik CJ. Knowledge, attitude, and practice toward contact isolation precautions among medical students in Shiraz, Iran. Am J Infect Control. 2006; 34(9): 593–6. DOI: 10.1016/j.ajic.2006.03.005 17097455

[9] Ibrahim AA and Elshafie SS. Knowledge, awareness, and attitude regarding infection prevention and control among medical students: a call for educational intervention. Adv Med Educ Pract. 2016; 7: 505–510. DOI: 10.2147/AMEP.S109830 27579002 PMC5001551

[10] Ogoina D, Pondei K, Adetunji B, Chima G, Isichei C and Gidado S. Knowledge, attitude and practice of standard precautions of infection control by hospital workers in two tertiary hospitals in Nigeria. J Infect Prev. 2015; 16(1): 16–22. DOI: 10.1177/1757177414558957 28989394 PMC5074133

[11] Alotaibi MM, Almasari SM, Alkadam AN, Alanazi YA and Al Gahtani KA. Knowledge and compliance with standard isolation precautions among healthcare students in Al-Kharj Governorate, Saudi Arabia. J Health Spec. 2017; 5: 162–70. DOI: 10.4103/jhs.JHS_94_16

[12] Suliman M, Aloush S, Aljezawi M and AlBashtawy M. Knowledge and practices of isolation precautions among nurses in Jordan. Am J Infect Control. 2018; 46(6): 680–684. DOI: 10.1016/j.ajic.2017.09.023 29103636 PMC7132704

[13] Amoran O and Onwube O. Infection control and practice of standard precautions among healthcare workers in Northern Nigeria. J Glob Infect Dis. 2013; 5: 156–63. DOI: 10.4103/0974-777X.122010 24672178 PMC3958986

[14] Osborne S. Influences on compliance with standard precautions among operating room nurses. Am J Infect Control. 2003; 31: 415–23. DOI: 10.1067/mic.2003.68 14639439

